# Fat binding capacity and modulation of the gut microbiota both determine the effect of wheat bran fractions on adiposity

**DOI:** 10.1038/s41598-017-05698-y

**Published:** 2017-07-17

**Authors:** Francesco Suriano, Laure B. Bindels, Joran Verspreet, Christophe M. Courtin, Kristin Verbeke, Patrice D. Cani, Audrey M. Neyrinck, Nathalie M. Delzenne

**Affiliations:** 1 Metabolism and Nutrition Research Group, Louvain Drug Research Institute, Université catholique de Louvain, B-1200 Brussels, Belgium; 2 Laboratory of Food Chemistry and Biochemistry, Leuven Food Science and Nutrition, Research Center (LFoRCe). KU Leuven, B-3001 Leuven, Belgium; 3 Translational Research Center for Gastrointestinal Disorders and Leuven Food Science and Nutrition Center, KU Leuven, B-3000 Leuven, Belgium; 4 Walloon Excellence in Life Sciences and BIOtechnology (WELBIO), Louvain Drug Research Institute, UCL, B-1200 Brussels, Belgium

## Abstract

The aim of this study was to determine the impact of different wheat bran fractions on the gut microbiota and fat binding capacity to explain their differential effects on metabolic and inflammatory disorders induced by a western diet (WD) in mice. Wheat bran derived arabinoxylan oligosaccharides (AXOS), a crude fraction of wheat bran (WB), or the same wheat bran with reduced particle size (WBs) were added to the WD of mice for 8 weeks. AXOS shifted the gut microbiota composition, blunted *Clostridium* and *Turicibacter* genera and strongly promoted *Bifidobacterium* and *Butyricicoccus* genera, independently of changes in gut antimicrobial peptide expression. AXOS was the most efficient to reduce adiposity. Only WB fraction promoted fat excretion and differed from the other fractions by the capacity to increase the *Akkermansia* genus and to counteract gut interleukin 1 beta (IL1β) overexpression. Strikingly, WBs promoted steatosis and adipose tissue inflammation, despite its ability -like WB- to increase bacterial diversity. In conclusion, wheat bran fractions differently affect metabolic and inflammatory disorders associated with WD feeding, depending on their particle size, their fat binding capacity and their influence on the gut microbiota. Those results might be useful to take into account in nutritional advices to control obesity.

## Introduction

In most Western countries, cereals and in particular wheat, represent a major source of dietary fibers. Dietary fibers are one of the most important classes of compounds in cereal grains related to positive health effects, and cereal whole grain products help reaching the recommended overall dietary fiber intake^1–3^. Wheat bran is the outermost layer of the wheat kernel, representing 14 to 19% of the total weight^4^. Wheat bran is a major source of dietary fiber, such as non-starch polysaccharides and lignin. Arabinoxylan (AX) is the most abundant dietary fiber in wheat bran^1, 5^ and accounts for 20 to 30% of dry wheat bran mass, or 70% of non-starch polysaccharides^4, 6^. It is a polymer with a D-xylose backbone linked with L-arabinose. Most AX in wheat bran are water insoluble because of crosslinking with neighboring units of cellulose, lignin, and proteins^5^. Since it is rich in insoluble fibers, wheat bran is beneficial in producing bulky stool and preventing colon cancer^7–10^. Wheat bran is also a low-cost byproduct of conventional wheat milling and a promising starting material for arabinoxylan oligosaccharides (AXOS) production^11^. AXOS are hydrolysis products of AX and are characterized by their lower average degree of polymerization and their average degree of arabinose substitution^5, 12^. Because of prehydrolysis, they are highly soluble and rapidly fermentable in the intestine^13^.

Gut microbiota is considered as an environmental factor involved in the control of body weight, metabolic alterations and inflammatory disorders occurring in obesity^14, 15^. AXOS derived from wheat bran, have been proposed as prebiotic nutrients prone to modulate the gut microbiota to restore host health^1, 16, 17^. Different mechanisms have been proposed to link the events occurring in the intestine following carbohydrate fermentation to the control of metabolic energy metabolism. Carbohydrate fermentation may promote the release of gastrointestinal peptides controlling food intake and/or insulin secretion (i.e., glucagon like peptide-1 (GLP-1), peptide YY (PYY), glucose-dependent insulinotropic peptide (GIP)). In addition, it has been shown that bacterial metabolites (short chain fatty acids (SCFA), conjugated linoleic acids) can act as regulators of adiposity (via G-protein-coupled receptors (GPR43) and/or peroxisome proliferator-activated receptor-γ (PPARγ)-dependent mechanisms)^18–20^. Finally, the changes in the activity of peptides/systems involved in the control of gut permeability (e.g., GLP-2, tight junction proteins such as zonula occludens-1 (ZO1) and occludin, endocannabinoid system) also contribute to modulate host metabolic alterations such as inflammation and endotoxemia^17, 21^.

Particle size influences the physiological effects of wheat bran such as colonic fermentation in humans^22^. Prehydrolysis of wheat bran AX into AXOS influences the latter even more^13^. SCFA production and fecal moisture are increased at the expense of other physiological effects such as delayed gastric emptying, increased mean transit time and increased stool weight when particle size decreases^23–25^. In addition, a recent study reported change in postprandial GLP‑1 response after consumption of wheat breads with different particle size in healthy men^26^. Experimental data show that specific changes in gut microbiota may be related to the improvement of adiposity by some wheat bran derivatives^27, 28^. However, to date, no study has applied a community-wide approach to evaluate if the changes in the gut microbiota play a role in the impact of wheat bran products differing by their physico-chemical properties -notably their particle size- on obesity and metabolic alterations induced by the western diet. The aim of this study was to determine the impact of different wheat bran fractions on the gut microbiota and fat binding capacity to explain their differential effects on metabolic and inflammatory disorders induced by a WD in mice.

## Results

### AXOS deeply influenced the gut microbiota composition without changing intestinal SCFA level, whereas WB and WBs induced differential and specific changes in gut microbes

**Figure 1:**
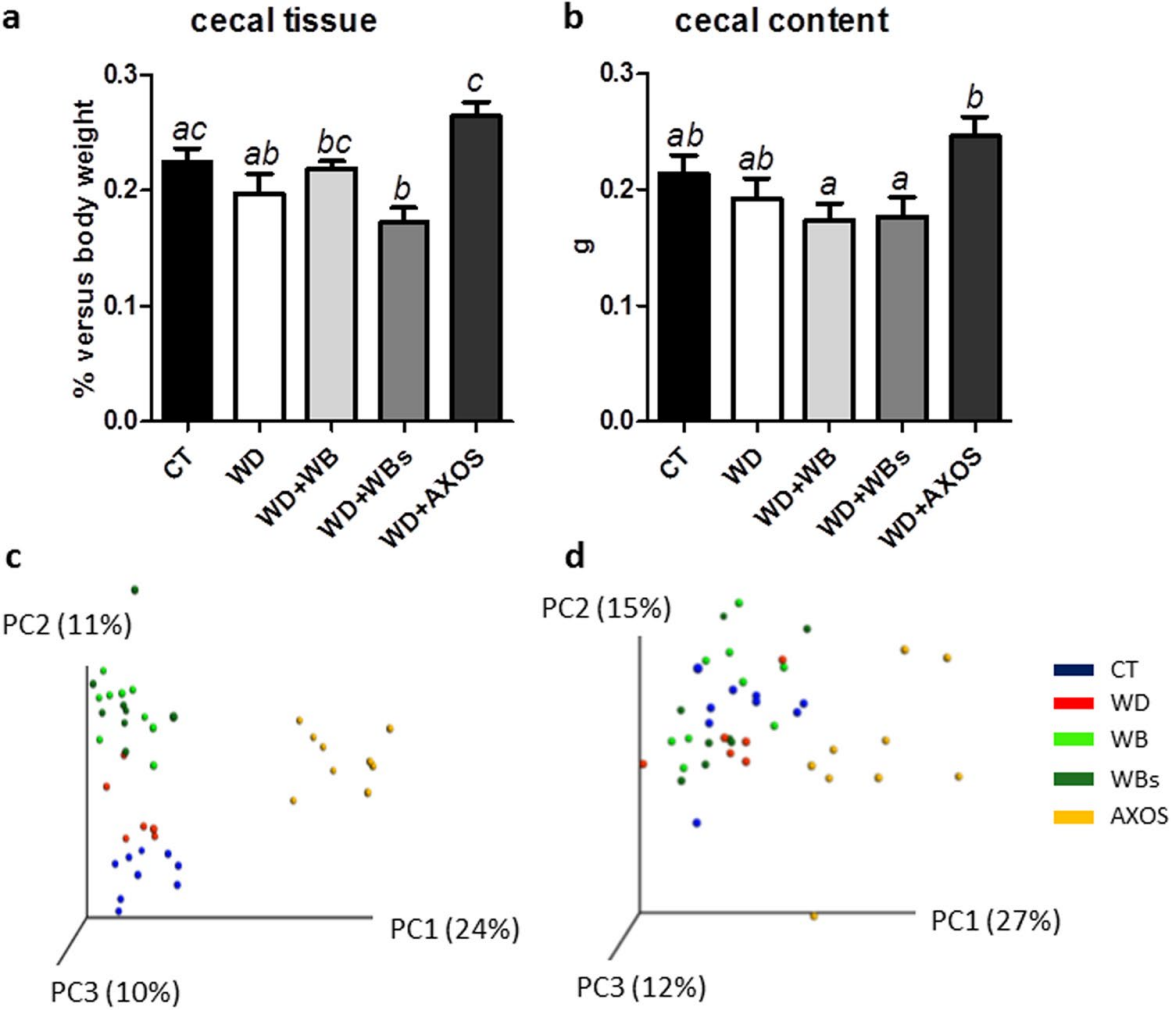
Impact of the three cereal fractions on the gut microbiota in the cecal content. Weight of cecal tissue (**a**), cecal content (**b**), Principal Coordinate Analysis plot of β-diversity based on Bray-Curtis distance (**c**) and Principal Coordinate Analysis plot of β-diversity based on Morista-Horn distance (**d**). Mice were fed a control diet (CT, blue), a western diet (WD, red), a WD supplemented with 5% of wheat bran fraction with large particles (WD+WB, clear green), a WD supplemented with 5% of wheat bran with reduced particle size (WD+WBs, dark green) or a WD supplemented with 5% of AXOS (WD+AXOS, yellow) for 8 weeks. Results are expressed as mean±SEM for **a** and **b** (n = 7–9). Data with different superscript letters are significantly different at p < 0.05 (ANOVA). Dietary treatments accounted for 39 and 48% of the variation in the dataset (Bray-Curtis and Morisita-Horn respectively, adonis method, 1000 permutations, p < 0.001).

Cecal tissue weight and the cecal content weight were not significantly modified by the western diet. AXOS increased the cecal tissue weight and the cecal content insert (significantly for the cecal tissue weight) versus the WD group (Fig. 1a,b). By contrast, no significant effect was observed for the WB and WBs fractions. SCFA reflecting bacterial fermentation were analysed in the cecal content using gas chromatography coupled to a mass spectrometer (Supplementary Fig. S1). No modification of cecal SCFAs levels was observed whatever the dietary treatment.

The composition of the gut microbiota in the cecal content was analyzed by Illumina sequencing of the 16S rRNA gene and qPCR.

#### Diversity indexes

Principal Coordinate Analysis (PCoA) of the beta-diversity indexes Bray-Curtis and Morisita-Horn revealed two major findings that were confirmed by further analyses at the taxa and OTU levels. First, WB and WBs fractions exerted a quite similar impact on the gut microbiota composition. Second, AXOS shifted away the composition of the gut microbiota and was the dietary treatment with the strongest impact on the gut microbiota composition (Fig. 1c,d). The impact of the WD on the gut microbiota composition was clear when using Bray-Curtis index, but not when using Morisita-Horn. Dietary treatments accounted for 39 and 48% of the variation in the dataset (Bray-Curtis and Morisita-Horn respectively, adonis method, 1000 permutations, p < 0.001) whereas cage effect accounted for 15% and 19% of the variation, indicating that the effect of the dietary treatment is the most important factor driving gut microbiota composition in our study. When comparing to the WD group, administration of both types of wheat bran fractions increased the alpha-diversity indexes for richness (significant effect of WB and WBs for observed species) and evenness (significant effect of WB and WBs for Shannon index), while administration of AXOS impacted mainly evenness (Supplementary Fig. S2).

#### Taxonomic analyses

**Figure 2:**
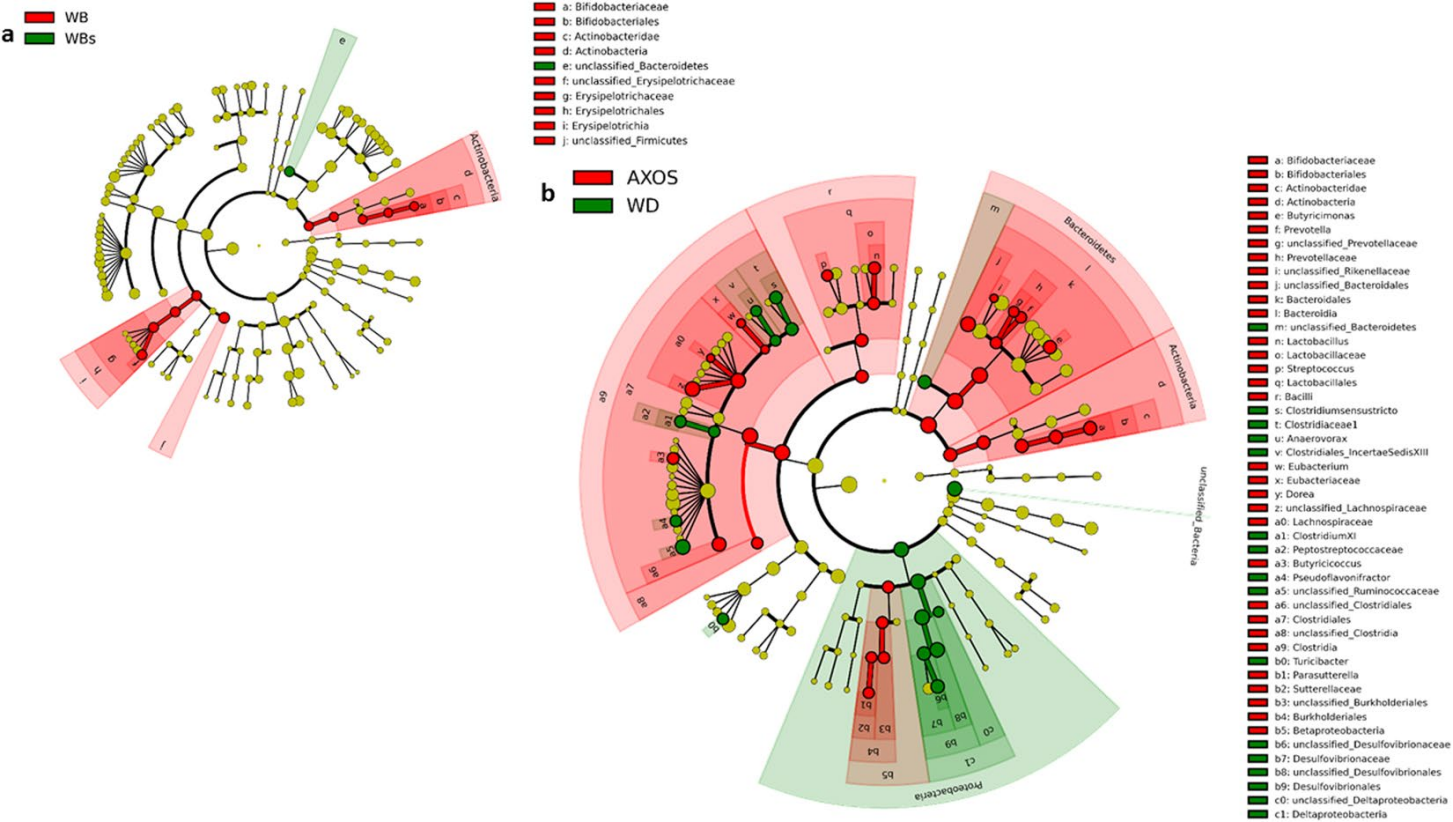
Discriminant analysis of the cecal microbiota using LEfSe. Mice were fed a control diet (CT), a western diet (WD), a WD supplemented with 5% of wheat bran fraction with large particles (WD+WB), a WD supplemented with 5% of wheat bran with reduced particle size (WD+WBs) or a WD supplemented with 5% of AXOS (WD+AXOS) for 8 weeks. Discriminant analysis between WB and WBs (**a**). Taxa enriched in the WB group are highlighted in red whereas taxa enriched in the WBs group are highlighted in green. Discriminant analysis between AXOS and WD (**b**). Taxa enriched in the AXOS group are highlighted in red whereas taxa enriched in the WD group are highlighted in green.

**Figure 3:**
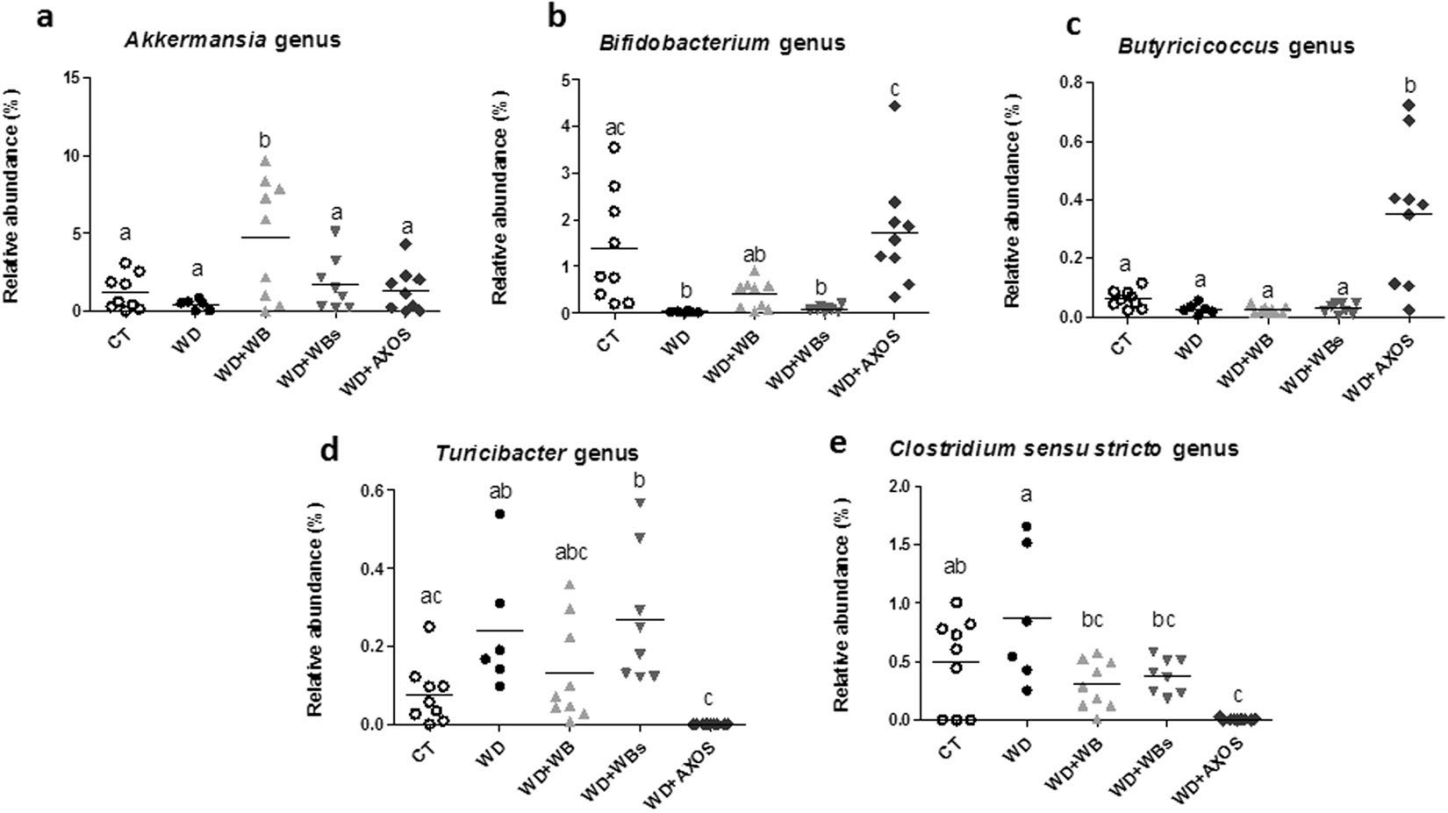
Impact of the three cereal fractions on the gut microbiota composition assessed by Illumina sequencing. Relative abundance of selected genera in cecal content of mice fed a control diet (CT), a western diet (WD), a WD supplemented with 5% of wheat bran fraction with large particles (WD+WB), a WD supplemented with 5% of wheat bran with reduced particle size (WD+WBs) or a WD supplemented with 5% of AXOS (WD+AXOS) for 8 weeks. Individual values are presented with mean. Data with different superscript letters are significantly different at *p* < 0.05 (ANOVA).

Microbes discriminant for dietary treatment were determined through a pairwise comparison using LEfSe (Fig. 2). For the WB-WBs comparison, bifidobacteria and unclassified *Erysipelotrichaceae* were identified as biomarkers of the WB group. Several bacteria were found to be discriminant for AXOS when compared to the WD groups, including the phylum Proteobacteria. Using parametric methods, *Akkermansia muciniphila* (and the higher level taxa from the same lineage) was the only taxon found to be different between WB and WBs at the q-level (Supplementary Fig. S3j and Supplementary Data S1). *Akkermansia muciniphila* levels were significantly increased by 11-fold in WB vs WD and not significantly increased by WBs (Fig. 3a). We obtained the same tendency with qPCR analysis (Supplementary Fig. S3a). AXOS changed the relative abundance of 25 bacterial taxa (Supplementary Data S1). One of the main changes related to a 57-fold increase for the *Bifidobacterium* genus and related family and order (Fig. 3b and Supplementary Data S1). This increase translated to a 16-fold increase at the *Actinobacteria* phylum level. In this set of experiment, bifidobacteria were decreased by the WD and their levels were fully restored by AXOS (Fig. 3b), as confirmed by qPCR (Supplementary Fig. S3b). Of note, a bifidogenic effect versus the WD group was also observed for the WB fraction (significantly only with the qPCR analysis) but it was absent for the WBs fraction (Fig. 3b and Supplementary Fig. S3b). Another important change was observed for *Butyricicoccus* (13-fold increase) after AXOS supplementation whereas *Turicibacter* disappeared (Fig. 3c), *Clostridium sensu stricto* was drastically reduced (113-fold decrease) (Fig. 3d) and *Desulfovibrionaceae* were lowered by half (Supplementary Data S1).

#### Phylogenetic analyses

Phylogenetic analyses revealed results similar to the taxonomic analyses. Among the 741 OTUs that were detected in the microbial ecosystem, 144 OTUs were significantly affected at the q-level (Supplementary Data S1). Most of these changes related to the impact of AXOS. Indeed, 78 OTUs were modified by the addition of AXOS in the WD. Six OTUs were increased more than a 100 fold, such as OTU 36 (*Bifidobacterium* sp., 1016-fold increase), OTU 83 (from the *Lachnospiraceae* family, 931-fold increase), OTU 90 (from the *Ruminococcaceae* family, 884-fold increase), OTU 100 (from the *Clostridiales* order, 593-fold increase), OTU 161 (from the *Lachnospiraceae* family, 181-fold increase) and OTU 35 (from the *Clostridiales* order, 162-fold increase) (Supplementary Fig. S3). Two OTUs (from the *Clostridiales* order) were decreased more than a 100-fold in presence of AXOS (OTU 173, 124-fold and OTU 75, 120-fold). When comparing the AXOS and WD groups, 14 OTUs were detected only in the AXOS group whereas 4 OTUs were detected only in the WD group. When comparing the WB and WBs groups, 13 OTUs showed different levels between dietary treatments. The most prominent differences are a 10-fold decrease in OTU 652 (*Allobaculum* sp. belonging to the *Erysipelotrichaceae* family) and a 3-fold decrease in OTU 8 (*Akkermansia muciniphila*) under WBs. The other 11 OTUs belong to the *Clostridiales* order, and were either increased or decreased by WBs by less than 6-fold. OTU 8 (*Akkermansia muciniphila*) and OTUs 37 and 138 (from the *Clostridiales* order) are the only 3 OTUs that were affected by the addition of WB in the WD but not by the addition of WBs to the same diet, if we exclude OTU 659 for which only 5 sequences were found in the all dataset.

### Several markers involved in gut homeostasis were not modified by the three cereal fractions

**Figure 4:**
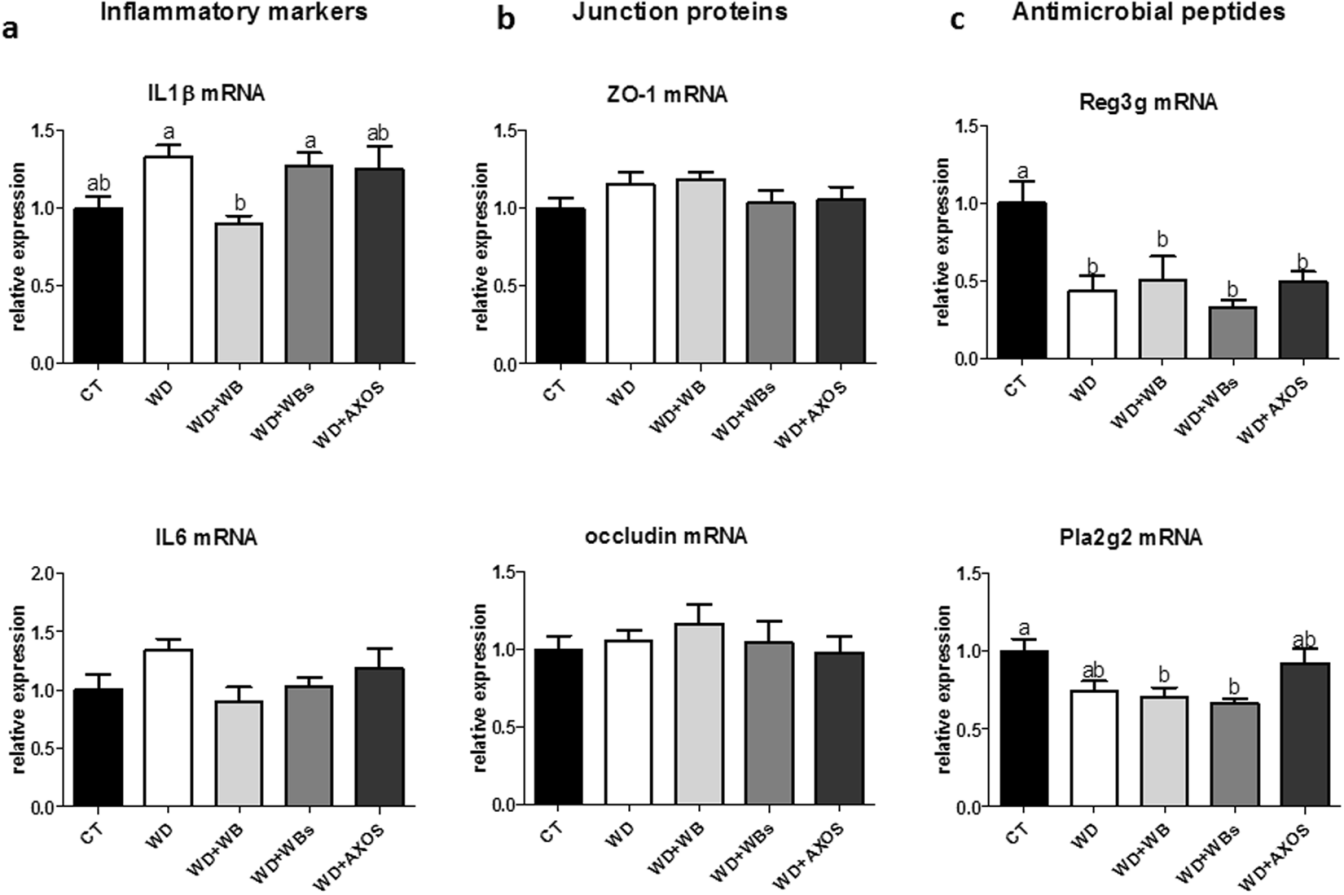
Impact of the three cereal fractions on gut barrier function. Expression of inflammatory markers (**a**), expression of junction proteins (**b**) and expression of secreting antimicrobial peptides (**c**) in the ileum of mice fed a control diet (CT), a western diet (WD), a WD supplemented with 5% of wheat bran (WD+WB), a WD supplemented with 5% of wheat bran with reduced particle size (WD+WBs) or a WD supplemented with 5% of AXOS (WD+AXOS) for 8 weeks. Results are expressed as mean±SEM (n = 7–9). Data with different superscript letters are significantly different at *p* < 0.05 (ANOVA).

Several markers involved in gut immune function and gut permeability were analysed both in the ileum (Fig. 4) and the colon. IL-1β and IL6 expression was slightly upregulated in the ileum by the WD as compared to the control mice without reaching significant p value. In addition, neither occludin expression, nor zonula occludens-1 (ZO1) expression were altered by the WD, suggesting that WD did not alter gut permeability (Fig. 4). WB fraction decreased the expression of IL1β in the ileum as compared to WD group and it is the sole significant effect of wheat bran materials observed on those gut inflammatory/permeability markers. We measured the expression of secreting antimicrobial peptides produced by epithelial cells in the ileum and the colon: phospholipase A2 group-II (PlA2g2) and C-type lectin, primarily the regenerating islet-derived 3-gamma (RegIIIγ) (Fig. 4). Although we observed significantly lower expression of RegIIIγ in the ileum segment due to the WD, none of the analysed antimicrobial peptides were affected by the supplementation with the cereal fractions (versus WD group) whatever the gut segments.

### AXOS, WB and WBs differently impact obesity and white adipose tissue

**Figure 5:**
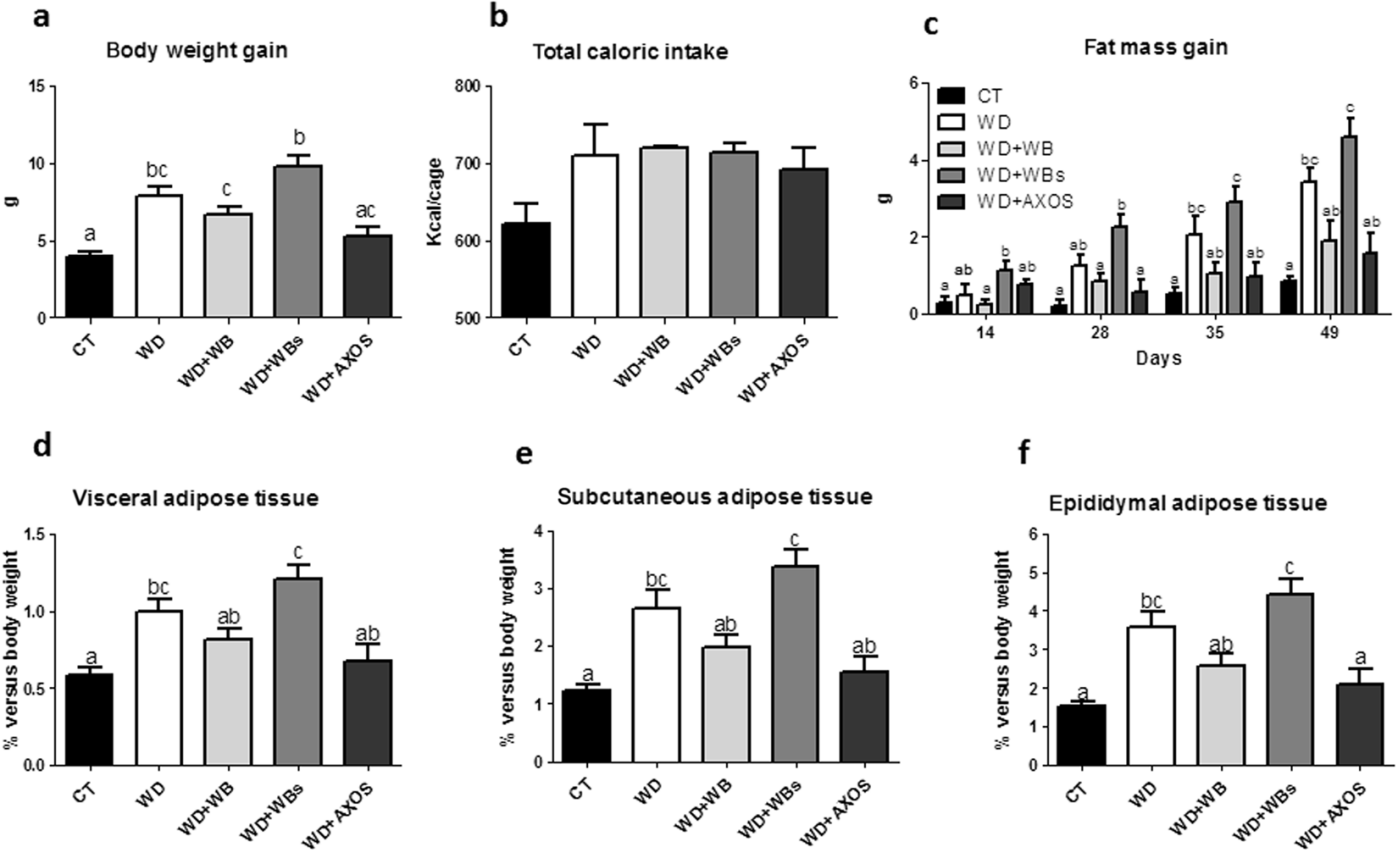
Impact of the three cereal fractions on obesity, dietary intake and fat mass development. Body weight gain (**a**), total caloric intake (**b**), fat mass gain measured by NMR at days 14, 28, 35 and 49 (**c**), weight of visceral adipose tissue (**d**), weight of subcutaneous adipose tissue (**e**), weight of epididymal adipose tissue (**f**). Mice were fed a control diet (CT), a western diet (WD), a WD supplemented with 5% of wheat bran fraction with large particles (WD+WB), a WD supplemented with 5% of wheat bran with reduced particle size (WD+WBs) or a WD supplemented with 5% of AXOS (WD+AXOS) for 8 weeks. Results are expressed as mean±SEM (n = 7–9 for **a**, **c**–**f** and n = 2–3 for **b**). Data with different superscript letters are significantly different at *p* < 0.05 (ANOVA).

The WD consumption increased the body weight gain and the adiposity compared to the control diet (Fig. 5a,c). Fat mass development due to the WD was confirmed by an increase of the visceral, subcutaneous and epididymal adipose tissues as compared with the control group (Fig. 5d–f). AXOS fraction supplementation reduced body weight gain and fat mass expansion without reaching significant p value except for the epididymal adipose tissue. Although wheat bran fractions WB and WBs had no significant effect on body weight gain and fat mass expansion due to WD, it is important to note that WBs group exhibited the higher values of body weight gain, fat mass gain and adiposity whereas wheat bran fraction with large particle (WB) tended to decrease body weight and adiposity whatever the type of adipose tissues. The differences between WB and WBs groups reached significant p value for all the parameters. However, the three cereal fractions did not modify significantly the total caloric intake (Fig. 5b). In accordance with the data relating the expansion of white adipose tissue, the portal levels of leptin, an adipokine produced proportionally to fat mass, were markedly increased in WD and WD+WBs groups as compared to the control group (Supplementary Fig. S4a). In contrast, the plasma levels of several gastrointestinal peptides regulating appetite that were secreted by the stomach (ghrelin), by the intestinal K-cells (GIP) or by the intestinal L-cells (GLP-1) were not significantly affected by the dietary treatments (Supplementary Fig. S4b–d).

**Figure 6:**
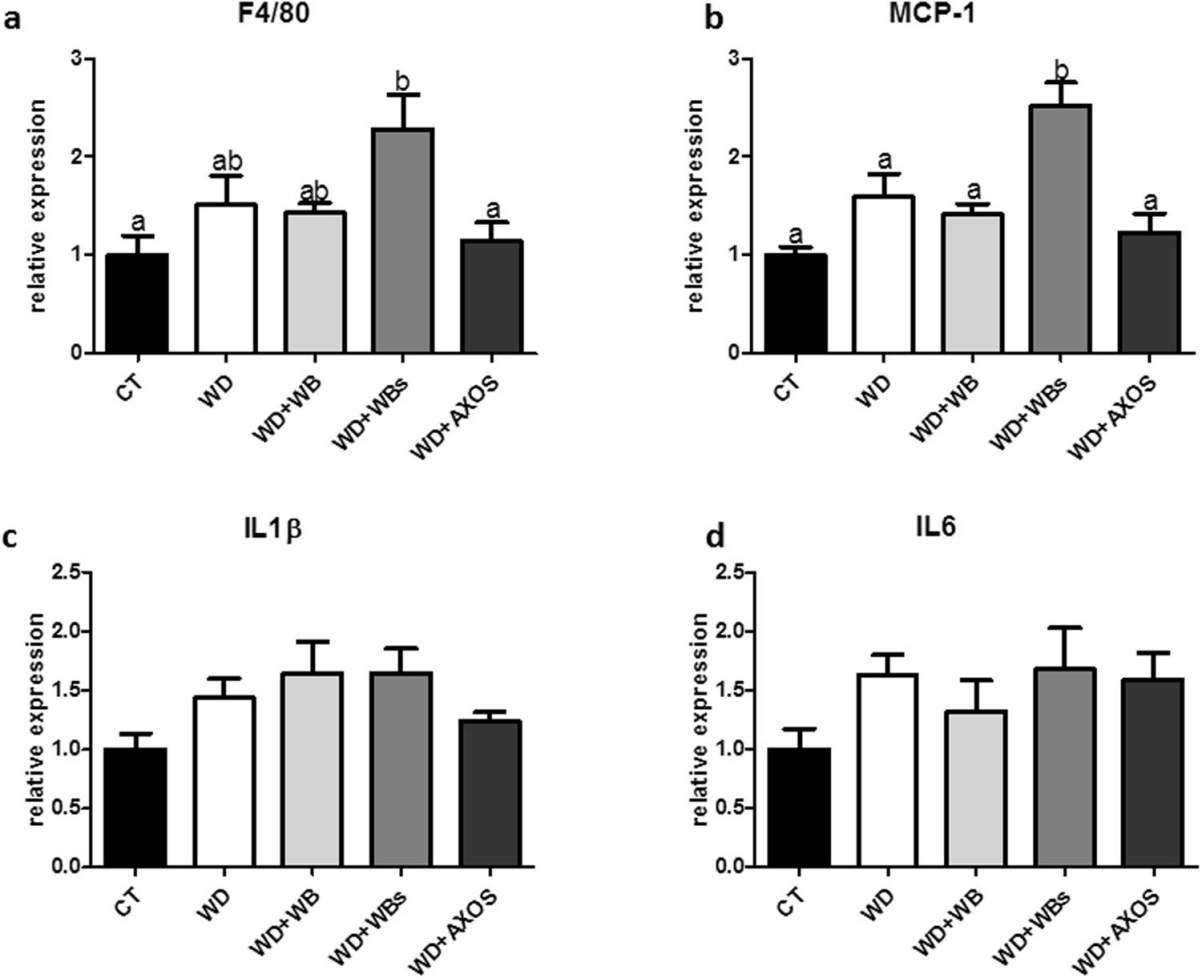
Inflammatory markers in visceral adipose tissue. Mice were fed a control diet (CT), a western diet (WD), a WD supplemented with 5% of wheat bran (WD+WB), a WD supplemented with 5% of wheat bran with reduced particle size (WD+WBs) or a WD supplemented with 5% of AXOS (WD+AXOS) for 8 weeks. Results are expressed as mean±SEM (n = 6–9). Data with different superscript letters are significantly different at *p* < 0.05 (ANOVA).

Key inflammatory markers were analysed by qPCR in the visceral adipose tissue (Fig. 6). WD did not affect the mRNA expression of inflammatory markers. By contrast and in accordance with the fat mass expansion, WD+WBs was the sole dietary treatment able to upregulate some of these markers (MCP-1 and F4/80) as compared to the WD group or CT group but also as compared to the WB group; this effect being significant for MCP-1 expression. The AXOS fraction and the crude WB fraction did not change significantly any of the four inflammatory markers analysed as compared to WD group.

### Wheat bran with reduced particle size (WBs) induced lipid accumulation in the liver

**Figure 7:**
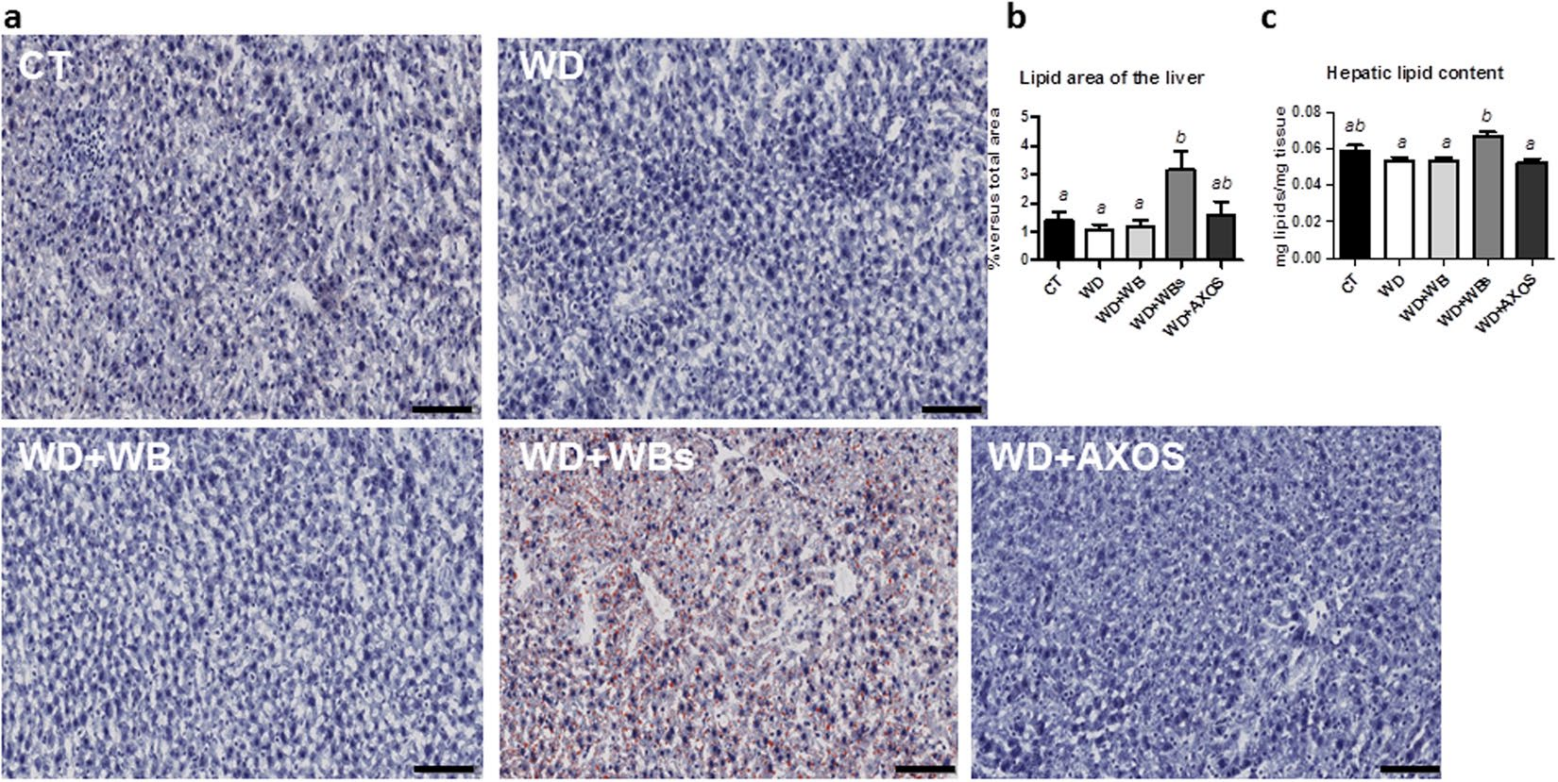
Impact of the three cereal fractions on lipid accumulation in the liver. Histochemical detection of neutral lipids of the liver sections (**a**), lipid area (% versus total area) (**b**) and lipid content of the liver (**c**) of mice fed a control diet (CT), a western diet (WD), a WD supplemented with 5% of wheat bran (WD+WB), a WD supplemented with 5% of wheat bran with reduced particle size (WD+WBs) or a WD supplemented with 5% of AXOS (WD+AXOS) for 8 weeks (scale bar = 100 μm). Results are expressed as mean±SEM (n = 7–9). Data with different superscript letters are significantly different at *p* < 0.05 (ANOVA).

**Figure 8:**
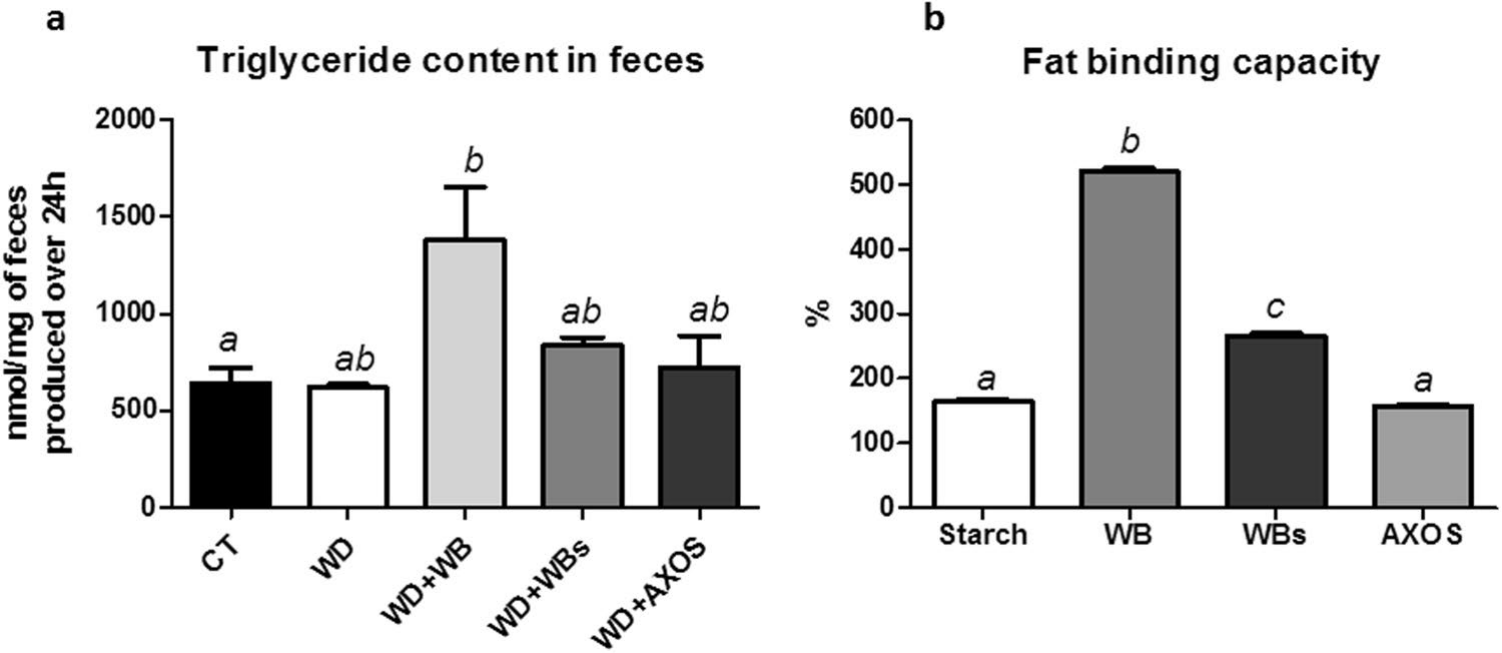
Impact of the three wheat bran fractions on triglyceride excretion in feces and fat binding capacity. Triglyceride content in feces (**a**) of mice fed a control diet (CT), a western diet (WD), a WD supplemented with 5% of wheat bran (WD+WB), a WD supplemented with 5% of wheat bran with reduced particle size (WD+WBs) or a WD supplemented with 5% of AXOS (WD+AXOS) for 8 weeks. *In vitro* fat binding capacity of starch and cereal fractions for soybean oil (**b**). Results are expressed as mean+SEM (n = 3 for **a** and n = 5 for **b**). Data with different superscript letters are significantly different at p < 0.05 (ANOVA).

Histological analysis after oil red O staining did not reveal steatosis in WD group as compared to CT group (Fig. 7a,b). Importantly, the wheat bran with reduced particle size (WBs) induced lipid accumulation as compared to control group or WD group whereas wheat bran with large particle size (WB) or AXOS did not exert any effect. Biochemical analysis of lipid content of the liver confirmed this result (Fig. 7c). Interestingly, in contrast to WBs fraction, WB supplementation leads to the higher excretion of triglycerides in the feces among all groups(Fig. 8a). This effect is probably linked to its higher fat binding capacity compared to the WBs or AXOS fractions (Fig. 8b).

### Changes in the gut microbiota composition induced by wheat bran fractions correlated with host parameters

**Figure 9:**
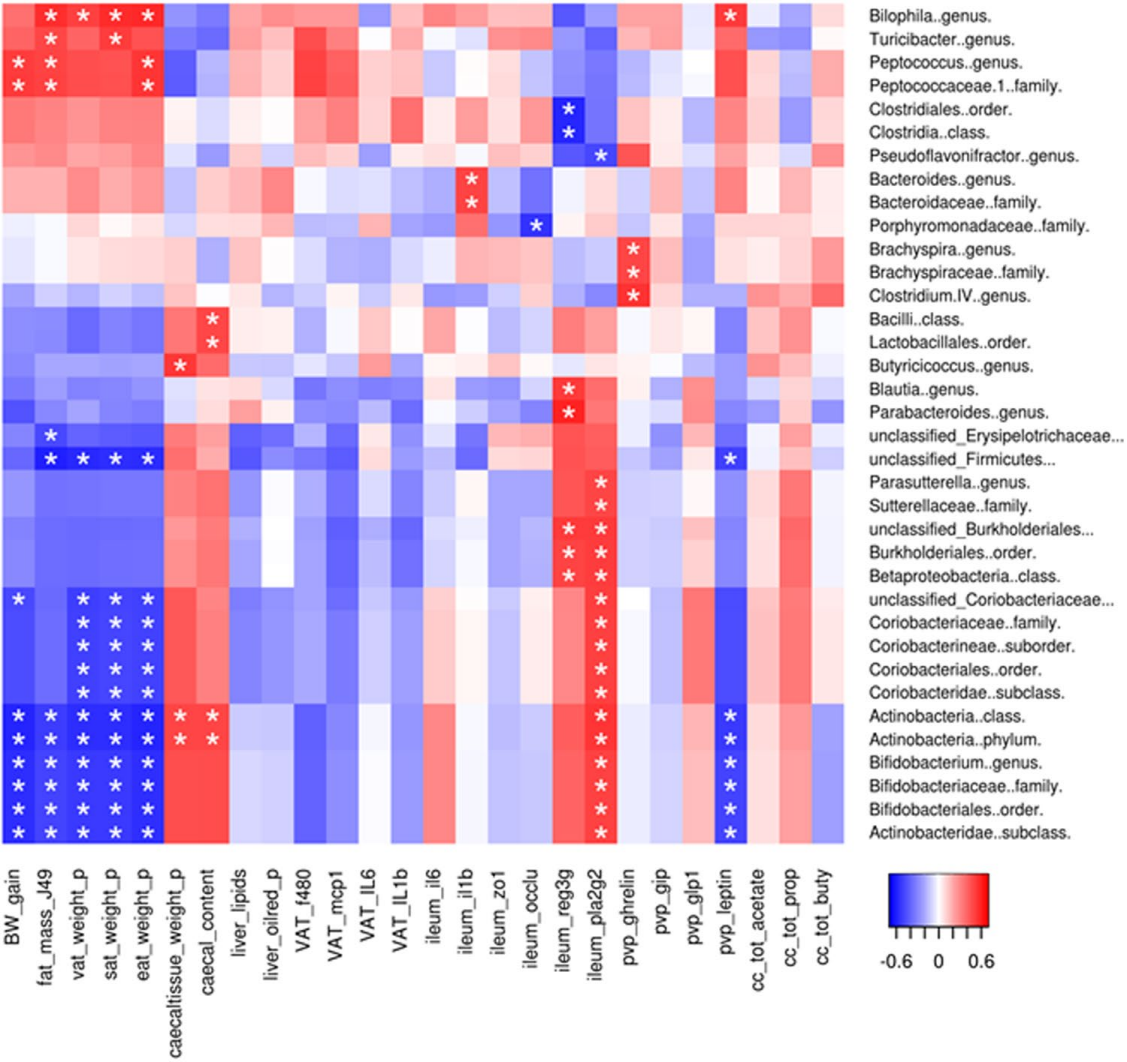
Correlations between bacterial taxa and host parameters. Pearson correlations were computed for all bacterial taxa with a relative abundance above 0.01% in at least one animal, and all measured host parameters. P values were adjusted for multiple testing according to the Bonferroni and Hochberg procedure. The color at each intersection refers to the value of the r coefficient; an asterisk indicates a significance correlation between these two parameters (adjusted p < 0.05). Only the bacterial taxa for which at least one significant correlation with a host parameter was detected, are displayed. Bacterial taxonomic level is indicated at the end of the name.

Multiple correlation analyses were performed to evaluate the potential links between gut microbial taxa and host metabolism and immunity among the five experimental groups (Fig. 9). Such analyses revealed a clear negative correlation between adipose tissue weights and two families from the Actinobacteria phylum, the *Coriobacteriaceae* and the *Bifidobacteriaceae*. The *Bifidobacterium* genus and its parent taxa were associated with a reduced body weight gain, reduced fat mass as assessed by NMR and reduced plasma leptin levels. In contrast, increased *Bilophila* levels were associated with increased fat mass, adipose tissue weights and plasma leptin levels. Finally, mRNA expression of PlA2g2 in the ileum was strongly positively correlated with the *Bifidobacteriaceae*, *Coriobacteriaceae* and *Sutterellaceae* families (and related parent taxa).

## Discussion

In the present study, we confirmed the prebiotic effect of AXOS produced from WB in a model of WD-induced obesity. Indeed, AXOS feeding increased bifidobacteria (demonstrated by qPCR and for the first time by sequencing of the 16S rRNA gene) in the cecal content of WD fed mice. This effect was associated with a reduction of epididymal fat expansion due to WD feeding confirming some of the results obtained in our previous study^27^. The lack of significant effect of AXOS on body weight gain may be related to the lower proportion of fat (45%) in the WD compared to the high fat (60%) diet used in previous study in which the body weight change due to HF diet was more drastic. Moreover, use of next-generation sequencing revealed other important microbial changes induced by AXOS. Sixty-one bacterial taxa were significantly affected by the dietary treatments despite the high inter-individual variation. No clear-cut separation between responders and non-responders were observed in our dataset, as it has been described with other fibers^29, 30^.Taxa related to bacteria associated with colitis and inflammatory disorders (*Turicibacter, ClostridiumProteobacteria*) or related to endotoxin-producing opportunistic pathogens (*Desulfovibrionaceae*) were reduced significantly, while those related to gut barrier protection (*Butyricicoccus*) increased^31, 32^. Correlation analysis revealed that higher abundancy of bifidobacteria was strongly associated with a reduced body weight gain, reduced fat mass as assessed by NMR and reduced plasma leptin levels. Although dietary treatments did not significantly modify its abundancy, it is worth to note that *Bilophila* genus proportion was negatively correlated to the fat mass, adipose tissue weights and plasma leptin levels in accordance with previous studies showing a link between *Bilophila wadsworthia*, the unique species described to date within this genus, and high fat diet-induced altered metabolism, inflammation and weight gain^33–36^.

One of the main objectives of our current work was to assess the potential relevance for host health of wheat bran and the importance of their particle size in relation to the modulation of the gut microbial composition in obese mice. Although AXOS and WB exhibited different fat binding capacities, those fractions exerted similar impacts on obesity and adiposity; a phenomenon that could be related to their influence on the gut microbiota^1, 14^. Indeed, a bifidogenic effect was observed upon supplementation with both the crude fraction of WB and the AXOS fraction. Importantly and in contrast to the AXOS fraction or the crude wheat bran (WB), wheat bran with reduced particle size (WBs) led to a highest body weight gain, fat mass gain and adiposity among the five groups. Accordingly, WBs increased the levels of adipo(cyto)kines in mice fed a WD as evidenced by the higher expression of inflammatory markers in visceral adipose tissue and higher leptinemia. Although the composition of the wheat bran fractions was not modified after particle size reduction, we observed important differences in metabolic effects after WB and WBs supplementation. This discrepancy may be the result of their different fat binding capacity (as shown *in vitro*) leading to fat leakage in fecal matter (as shown *in vivo* by a higher proportion of fecal triglycerides) as already mentioned for two distinct fermentable carbohydrates targeting the gut microbiota (long chain arabinoxylans and inulin-type fructans)^37^. The different fat binding capacity between the fractions could be also one of the mechanisms explaining the difference in ectopic lipid accumulation in the liver tissue, which could result from a decrease in the pool of dietary non esterified fatty acids redistributed from the adipose tissue to the liver. However, we may not exclude that different effects observed with the WB fractions on adiposity resulted from their intrinsic capacity to modulate gut microbiota. For example, when comparing the WB and WBs groups, one of the most prominent differences is an increase of *Akkermansia muciniphila* under WB. Our recent data obtained in high fat diet fed mice have shown that *Akkermansia muciniphila* administrated through daily gavage is able to increase the fecal energy excretion and could therefore, participate to the control of adiposity^38^. Furthermore, a bifidogenic effect was observed only with the crude fraction, as already shown in a previous study only after 3 weeks of supplementation^39^. In fact, both taxa were identified as key players in the cross-talk between gut microbiota and host metabolism particularly in the context of obesity^40^. Gut microbiota may contribute to energy metabolism through the production of SCFA by colonic fermentation and *in vitro* studies reported changes in their proportions between large particle wheat bran and small particle wheat bran^41, 42^. It is generally admitted that the fermentation of AXOS is able to promote butyrate production^43^. However, in our study, cecal contents of butyrate, propionate and acetate were not significantly modified whatever the dietary treatments. We may not rule out that an increased production of butyrate (or other SCFA) could occur, but the fact that the SCFA produced may be absorbed, or excreted, which would not allow to point out changes in their mean level in the intestinal content. Probably, in our context, difference in SCFA cannot explain the difference in phenotype.

We were unable to highlight inflammatory disorders in the visceral adipose tissue or the ileum in WD mice as compared to control mice. The lack of significant inflammatory disorders between WD group versus CT group -together with the lack of effect on parameters related to gut permeability (expression of 2 key tight junction proteins)- may be related to the lower proportion of fat (45%) in the WD compared to the high fat (60%) diet used in previous studies (for review see ref. 44). We have analyzed antimicrobial peptides that are produced by the host and contribute to shape the composition of the gut microbiota. These peptides participate to the gut barrier function and are an attractive mechanism for the modulation of the gut ecosystem by nutrients^45, 46^. Among them, the expression of RegIIIγ, a key host factor controlling microbiota composition and involved in gut immunity, was lower in the ileum of WD mice as compared to control mice as previously shown in a diet induced obesity model using high fat diet (60%)^40, 46^. Interestingly, an anti-inflammatory effect of wheat bran was evidenced by a significantly lower expression of IL1β in the ileum only for the unmodified crude fraction (WB). This effect seemed to be independent of the gut barrier functions as the expression of tight junction proteins (ZO1 and occludin) and of the antimicrobial peptide RegIIIγ was not affected in contrast to what is observed for fructan-type prebiotics^40^. Correlation analysis revealed that the ileal expression of Il1β was positively correlated to the *Bacteroides* genus although this genus was not significantly affected by the dietary treatments. The usual limitations linked to individual experimental animals also apply to this study^47^. Only one gender and one genetic background were tested. In our case, we controlled for cage effects.

In conclusion, wheat bran fractions differently affect metabolic and inflammatory disorders associated with WD feeding. Particle size can determine health effects of wheat bran in the context of obesity. In addition, differences in metabolic response of the different fractions also depend on their fat binding capacity and gut microbiota modulating properties (namely *Akkermansia* and bifidobacteria). Indeed, only the crude fraction of wheat bran promoted fat excretion and distinguished itself from the other fractions by the capacity to increase the *Akkermansia* genus and to counteract gut IL1β overexpression. Moreover, our findings confirmed the prebiotic potential of AXOS. Although AXOS and WB exhibited different fat binding capacities, those fractions exerted similar impacts on adiposity. This phenomenon could be related to their influence on the gut microbiota.

## Materials and Methods

### Ethics Statement

All experiments were performed in strict accordance with relevant guidelines and regulations for the care and use of animals. All mouse experiments were approved by and performed in accordance with the guidelines of the local ethics committee for animal care of the Health Sector of the Université catholique de Louvain under the supervision of Prof. F. Lemaigre and Prof. JP Dehoux and under the specific agreement numbers 2014/UCL/MD/022. Housing conditions were as specified by the Belgian Law of 29 May 2013, on the protection of laboratory animals (Agreement LA 1230314). Every effort was made to minimize animal pain, suffering, and distress and to reduce the number of animals used.

### Animals, diets and experimental setup

Forty-five male C57BL6 mice (9 weeks old at the beginning of the experiment, Janvier laboratories, France) were housed in groups of 3 per cage in a controlled environment (12-hour daylight cycle) with free access to food and water. After one week of acclimatisation, mice were divided in 5 groups (n = 9/group): a control group (CT), fed with a control diet (D12450K, Research Diet®, 10% fat, 70% carbohydrates in kcal/g), a group fed with a WD (D12451, Researcher Diet®, 45% fat, 35% carbohydrates in kcal/g), a group fed the WD, supplemented with 5% unmodified wheat bran (1690 µm, WD+WB group), a group fed the WD, supplemented with 5% wheat bran with reduced particle size (150 µm, WD+WBs group) and a group fed the WD, supplemented with 5% wheat bran derived arabinoxylan oligosaccharides (WD+AXOS group). The composition of the wheat bran materials has been detailed in Supplementary Table S1. Food intake and water intake were recorded twice a week. The total caloric intake was obtained by multiplying total food intake (g) for 3 mice per cage (n = 3) by the caloric value of the diets, i.e. 3.85 kcal/g, 4.73 kcal/g for CT and WD diets respectively. The caloric value of the WD supplemented with cereal fractions was calculated taking in to account that these diets were composed of 95% WD diet and 5% fractions considering that those fractions were completely processed by the gut microbiota into SCFA available to the host and that the maximal caloric intake through this process would be 2 kcal/g on average (value established by the commission directive 2008/100/EC). The total caloric intake results are presented for the whole study and represent the sum of the diet consumed during 8 weeks for three mice. Total fat mass was determined using a 7.5 MHz Time domain-Nuclear magnetic resonance (LF50 minispec, Bruker, Germany). After 8 weeks of dietary treatment and a 6-hour period of fasting, mice were anesthetised with isoflurane (Forene®, Abbott, Queenborough, Kent, England) before exsanguination and tissue sampling. Mice were killed by cervical dislocation. Portal blood was taken in <30 sec and directly flushed within tubes containing dipeptidyl peptidase IV (DPPIV) inhibitor (Millipore, St Charles, MO, USA) and a cocktail of general protease inibitors containing EDTA, bestatin, leupeptin, aprotinin, E-64 and 4-(2-aminoethyl)-benzenesulfonyl fluoride hydrochloride (P2714-Sigma protease cocktail, Sigma, Saint Louis, MO, USA and Roche Pefabloc SC, Roche Diagnostics, Vilvoorde, Belgium). Plasma was immediately collected after centrifugation and stored at −80 °C in tubes containing the same protease inhibitors as those used for gut peptide determination. Liver, white adipose tissues (visceral, epididymal and inguinal subcutaneous), and gut segments (from ileum, proximal colon and caecum) were carefully dissected, weighed and immersed in liquid nitrogen before storage at −80 °C.

### Biochemical analysis

Portal concentrations of GLP-1, GIP, ghrelin and leptin were determined in 2 × 15 µl of plasma using a multiplex immunoassay kit (Bioplex, Bio-Rad) and measured using Luminex technology (Bioplex, Bio-Rad). Lipid content was measured in the liver tissue after extraction with chloroform–methanol according to the Folch method^48^. Triglycerides were measured in feces after extraction with chloroform-methanol. Briefly, 100 mg of dried powder-reduced feces were homogenised in 2.6 ml of chloroform: methanol (2:1). The homogenate was filtered using a Whatman filter placed at the end of a syringe, and recovered in a 15 ml falcon tube. The filtrate was washed up three times with phosphate buffer (pH 7.4). The chloroform phase was evaporated under nitrogen flux and the dried residue was solubilised in 800 µl of isopropanol. Triglycerides concentration was measured using a kit coupling an enzymatic reaction and spectrophotometric detection of the final product (Diasys Diagnostic and System, Holzheim, Germany). All samples were run in duplicate.

### Fat binding assay

Fat binding capacity (FBC) of wheat fractions was initially carried out by weighing a centrifuge tube containing 0.5 g of sample, adding 10 ml of soybean oil, and mixing on a vortex mixer for 1 min to disperse the sample. The contents were left at ambient temperature for 30 min with shaking for 5 seconds every 10 min and then centrifuged at 2500 g for 25 minutes. After the supernatant was removed, the tube was weighed again. FBC was calculated as follows: FBC (%) = [fat bound (g)/sample weight (g)] × 100^49^.

### Fat histochemical detection

A fraction of the main liver lobe was fixed-frozen in Tissue-tek in liquid nitrogen-cold isopentane. For the detection of neutral lipids, frozen sections were sliced and stained with the oil red O, using 0.5% oil red O dissolved in propylene glycol for 10 min at 60 °C. The sliced sections were then counterstained with a Hemalum solution. The lipid area was analysed as previously described in Neyrinck *et al*.^50^.

### Real-time quantitative PCR

Total RNA was isolated from tissues using the TriPure isolation reagent kit (Roche Diagnostics, Penzberg, Germany). For adipose tissue, RNA integrity number (RIN) was calculated using a Agilent 2100 Bioanalyzer (Agilent Technologies, Santa Clara, CA); samples were selected only when their RIN value was above 6. Complementary DNA was prepared by reverse transcription of 1 μg total RNA using the Kit Reverse Transcription System (Promega,Madison,WI). Real-time polymerase chain reaction (PCR) was performed with a CFX96 Touch Real-Time PCR Detection System and software (Biorad Laboratories Ltd, UK) using SYBR Green (Applied Biosystems and Eurogentec, Verviers, Belgium) for detection. All samples were run in duplicate in a single 96-well reaction plate, and data were analyzed according to the 2-ΔΔCT method. The purity of the amplified product was verified by analyzing the melting curve performed at the end of amplification. The ribosomal protein L19 (RPL19) gene was chosen as a reference gene. Primer sequences are presented in Supplementary Table S2.

### Short chain fatty acids analysis

SCFA levels in cecal content were analyzed using gas chromatography coupled to a mass spectrometer (GC-MS). Cecal aliquots of 60 mg were suspended in 1 ml saturated NaCl and 2-ethylbutyrate (50 µL; 1.25 mg/mL) was added as internal standard. Samples were shortly vortexed after which 3 mL of diethylether (Sigma) and 150 µL of sulfuric acid were added to the sample. After centrifugation, the ether layer was separated and dried over anhydrous Na_2_SO_4_. The short-chain fatty acids (SCFAs) were analyzed on a GC-MS system (Trace GC, Thermoquest, Rodano, Italy and DSQ II, Thermo Electron, San Jose, USA) equipped with a Stabilwax DA column (30 m × 0.25 mm ID, 0.25 µm film thickness; Interscience, Belgium). The initial oven temperature of 40 °C was held for 3 min and ramped with 4 °C/min to 140 °C and with 16 °C/min to 240 °C. Acetate, propionate and butyrate were quantified with appropriate calibration curves obtained using internal standard quantitation.

### Analysis of the gut microbiota

Genomic DNA was extracted from the cecal content using a QIAamp DNA Stool Mini Kit (Qiagen, Germany), including a bead-beating step. The composition of the gut microbiota was analysed by Illumina sequencing of the 16S rRNA gene and qPCR. Absolute quantification of *Bifidobacterium spp*. and *Akkermansia muciniphila* was performed by quantitative real-time PCR (qPCR) (primers presented in Supplementary Table S2) as previously described^51^. The V5-V6 region of the 16S rRNA gene was amplified by PCR with modified primers^52^. The amplicons were purified, quantified and sequenced using an Illumina Miseq to produce 2 × 300-bp sequencing products at the University of Minnesota Genomics Center. Initial quality-filtering of the reads was conducted with Illumina Software, yielding an average of 67765 pass-filter reads per sample. Quality scores were visualized, and reads were trimmed to 220 bp (R1) and 200 bp (R2). The reads were merged with the merge-Illumina-pairs application^53^. For all samples but one, a subset of 25000 reads was randomly selected using Mothur v.1.25.0^54^ to avoid large disparities in the number of sequences. Subsequently, the UPARSE pipeline implemented in USEARCH v7.0.1001^55^ was used to further process the sequences. Putative chimaeras were identified against the Gold reference database and removed. Clustering was performed with a 98% similarity cut-off to designate operational taxonomic units (OTUs). Non-chimeric sequences were also subjected to taxonomic classification using the RDP MultiClassifier 1.1 from the Ribosomal Database Project^56^ for phylum to genus characterization of the cecal microbiome. The phylotypes were computed as percent proportions based on the total number of sequences in each sample. Full protocol, detailed statistical analysis and accession numbers are provided in the Supplemental materials.

### Statistical analysis

Data are presented as mean ± SEM. Statistical significance between groups was assessed by one-way ANOVA. Dixon’s Q-test was performed to remove outliers (95% confidence level). The statistically significant ANOVA tests were followed by post hoc Tukey’s multiple comparison tests using GraphPad Prism software (version 5.00, GraphPad Software, San Diego, California, USA). Data with different superscript letters were significantly different (p ≤ 0.05) according to the post hoc ANOVA statistical analysis. Multiple correlation analyses were computed and visualized in R using Pearson correlation method in a personal script, with p values being adjusted for multiple testing according to the Benjamini and Hochberg procedure^57^; p ≤ 0.05 was considered as statistically significant.

## Electronic supplementary material

Supplementary Information

Dataset 1
