# Supplementary material for: Fat binding capacity and modulation of the gut microbiota both determine the effect of wheat bran fractions on adiposity
Source: Sci Rep. 2017 Jul 17;7:5621. doi: 10.1038/s41598-017-05698-y (PMC5514075; doi:10.1038/s41598-017-05698-y)
Supplement: Supplementary file 1 — Supplementary Information [file 41598_2017_5698_MOESM1_ESM.pdf]

Supplementary Information

Title: Fat binding capacity and modulation of the gut microbiota both determine the effect of wheat bran fractions on adiposity

Francesco Suriano[1,*], Laure B. Bindels[1,*], Joran Verspreet[2], Christophe M. Courtin[2], Kristin Verbeke[3], Patrice D. Cani[1,4], Audrey M. Neyrinck[1], Nathalie M. Delzenne[1,+]

[1]Metabolism and Nutrition Research Group, Louvain Drug Research Institute, Université catholique de Louvain, B-1200 Brussels, Belgium.

[2]Laboratory of Food Chemistry and Biochemistry, Leuven Food Science and Nutrition Research Center (LFoRCe). KU Leuven, B-3001 Leuven, Belgium

[3]Translational Research Center for Gastrointestinal Disorders and Leuven Food Science and Nutrition Center, KU Leuven, B-3000 Leuven, Belgium.

[4]Walloon Excellence in Life sciences and BIOtechnology (WELBIO), Louvain Drug Research Institute, UCL, B-1200 Brussels, Belgium.

*These authors contributed equally to this work

[+]corresponding nathalie.delzenne@uclouvain.be

Supplemental materials: next-generation sequencing of DNA extracted from the caecal content

Amplicon sequencing of the caecal microbiome was done at the University of Minnesota Genomics Center, as previously described[1,2] .The V5-V6 region of the 16S rRNA gene was PCR-enriched using the primer pair 784F (5'-RGGATTAGATACCC-3') and 1064R (5'-CGACRRCCATGCANCACCT-3') in a 25 μl PCR reaction containing 5 μl of template DNA, 5 μl of 2X HotStar PCR master mix, 500 nM of final concentration of primers and 0.025 U/μl of HostStar Taq+ polymerase (QIAGEN). PCR-enrichment reactions were conducted as follows, an initial denaturation step at 95°C for 5 min followed by 20-25 cycles of denaturation (50 s at 94°C), annealing (30 s at 40°C), and elongation (30 s at 72°C)[1-4].

Next, the PCR-enriched samples were diluted 1:100 in water for input into library tailing PCR. The PCR reaction was analogous to the one conducted for enrichment except with a Taq polymerase concentration of 0.25 U/ μl, while the cycling conditions used were as follows, initial denaturation at 95°C for 5 min followed by 10-15 cycles of denaturation (50 s at 94°C), annealing (30 s at 40°C), and elongation (1 min at 72°C). The primers used for tailing are the following : F-indexing primer AATGATACGGCGACCACCGAGATCTACAC[i5]TCGTCG GCAGCGTC and R-indexing primer CAAGCAGAAGACGGCATACGAGAT[i7]GTCTCG TGGGCTCGG, where [i5] and [i7] refer to the index sequence codes used by Illumina.

The resulting PCR products were quantitated by PicoGreen (Life Technologies). A subset of the amplicon libraries were spot-checked on a Bioanalyzer High-Sensitivity DNA Chip (Agilent Technologoies) for correct amplicon size. Next, samples were normalized to 2nM and pooled together. The total volume of the libraries was reduced by SpeedVac and amplicons were size-selected at 420 bp +/- 20% using the Caliper XT (Perkin Elmer). Next, library pools were cleaned-up by 1.8X AMPureXP beads (Beckman Coulter) and eluted in water. The final pool was quantitated by PicoGreen and normalized to 2 nM for input into Illumina MiSeq (v3 Kit) to produce 2x300 bp sequencing products. Clustering was done at 10 pM with a 5% spike of PhiX. Initial quality filtering of the reads was performed with the Illumina Software, yielding an average of 133532 pass-filter reads per sample. Quality scores were visualized with the FastQC software (http://www.bioinformatics.babraham.ac.uk/publications.html), and reads were trimmed to 220 bp (R1) and 200 bp (R2) with the FASTX-Toolkit (http://hannonlab.cshl.edu/fastx_toolkit/). Next, reads were merged with the merge-illumina-pairs application v1.4.2 (with P = 0.03, enforced Q30 check, perfect matching to primers which are removed by the software, and otherwise default settings including no ambiguous

nucleotides allowed)[5]. For all samples but one, a subset of 25000 reads was randomly selected using Mothur v1.25.0 [6], to avoid large disparities in the number of sequences. One sample with 21948 quality-controlled merge reads was not subsampled.

Subsequently, the UPARSE pipeline implemented in USEARCH v7.0.1001[7] was used to further process the sequences. Putative chimeras were identified against the Gold reference database and removed. Clustering was performed with 98% similarity cutoff to designate Operational Taxonomic Units (OTUs). A representative sequence of each OTU was used for taxonomy assignment with the RDP classifier using assign_taxonomy.py in QIIME[8]. Non-chimeric sequences were also subjected to taxonomic classification using the RDP MultiClassifier 1.1 from the Ribosomal Database Project [9] , for phylum to genus characterization of the caecal microbiome.

The phylotypes were computed as percent proportions based on the total number of sequences in each sample. Alpha diversity indexes, beta diversity indexes and Adonis p-values were calculated using QIIME [8]. PCoA plot of the beta-diversity indexes were obtained using EMPeror [10].

Significantly affected taxa and OTUs were identified by one-way ANOVA followed by Tukey post-tests using R. Discriminant microbes were identified using LEfSe [11]. The p-value of the one-way ANOVA was adjusted (q-values) to control for the false discovery rate (FDR) for multiple tests according to the Benjamini and Hochberg procedure [12].

The sequences used for analysis can be found in the MG-RAST database under the project name “Branding” (ID 17584), with the following accession numbers: 4693022.3 to 4693070.3.

References

1 Bindels, L. B. *et al.* Synbiotic approach restores intestinal homeostasis and prolongs survival in leukaemic mice with cachexia. *ISME J* **10**, 1456-1470, doi:10.1038/ismej.2015.209 (2016).

2 Simon, M. C. *et al.* Intake of Lactobacillus reuteri improves incretin and insulin secretion in glucose-tolerant humans: a proof of concept. *Diabetes Care* **38**, 1827-1834, doi:10.2337/dc14-2690 (2015).

3 Bindels, L. B. *et al.* Resistant starch can improve insulin sensitivity independently of the gut microbiota. *Microbiome* **5**, 12, doi:10.1186/s40168-017-0230-5 (2017).

4 Claesson, M. J. *et al.* Comparison of two next-generation sequencing technologies for resolving highly complex microbiota composition using tandem variable 16S rRNA gene regions. *Nucleic Acids Res* **38**, e200, doi:10.1093/nar/gkq873 (2010).

5 Eren, A. M., Vineis, J. H., Morrison, H. G. & Sogin, M. L. A filtering method to generate high quality short reads using illumina paired-end technology. *PLoS One* **8**, e66643, doi:10.1371/journal.pone.0066643 (2013).

6 Schloss, P. D. *et al.* Introducing mothur: open-source, platform-independent, community-supported software for describing and comparing microbial communities. *Appl Environ Microbiol* **75**, 7537-7541, doi:10.1128/AEM.01541-09 (2009).
7 Edgar, R. C. UPARSE: highly accurate OTU sequences from microbial amplicon reads. *Nat Methods* **10**, 996-998, doi:10.1038/nmeth.2604 (2013).
8 Caporaso, J. G. *et al.* QIIME allows analysis of high-throughput community sequencing data. *Nat Methods* **7**, 335-336, doi:10.1038/nmeth.f.303 (2010).
9 Cole, J. R. *et al.* Ribosomal Database Project: data and tools for high throughput rRNA analysis. *Nucleic Acids Res* **42**, D633-642, doi:10.1093/nar/gkt1244 (2014).
10 Vazquez-Baeza, Y., Pirrung, M., Gonzalez, A. & Knight, R. EMPeror: a tool for visualizing high-throughput microbial community data. *Gigascience* **2**, 16, doi:10.1186/2047-217X-2-16 (2013).
11 Segata, N. *et al.* Metagenomic biomarker discovery and explanation. *Genome Biol* **12**, R60, doi:10.1186/gb-2011-12-6-r60 (2011).
12 Benjamini, Y. & Hochberg, Y. Controlling the False Discovery Rate: A Practical and Powerful Approach to Multiple Testing. *Journal of the Royal Statistical Society. Series B (Methodological)* **57**, 289-300 (1995).

Supplementary Table 1: Characterization of wheat bran materials

The average ± standard deviation is shown. Analyses were performed at least in duplicate.

Constituent	Wheat bran (WB)	Wheat bran with reduced particle size (WBs)
Dietary fiber content (% dm)	53.5 ± 0.5	54.4 ± 0.2
Arabinoxylan (AX) content (% dm)	27.0 ± 1.4	26.8 ± 1.0
Water extractable AX content (% dm)	0.74 ± 0.01	1.01 ± 0.04
A/X ratio	0.58 ± 0.03	0.59 ± 0.01
β-Glucan content (% dm)	2.3 ± 0.1	2.5 ± 0.1
Non-cellulose glucose content (% dm)	15.9 ± 0.9	15.0 ± 0.1
Protein content (% dm)	19.0 ± 0.3	17.4 ± 0.1
Ash content (% dm)	6.2 ± 0.1	6.3 ± 0.1
Extractable lipid content (% dm)	3.6 ± 0.1	5.5 ± 0.5

AX, Arabinoxylan; A/X ratio, arabinose/xylose ratio; dm, dry matter.

Arabinoxylan oligosaccharides (AXOS)	
Arabinoxylan oligosaccharides (AXOS) content (% dm)	80.2
of which xylo-oligosaccharides (XOS DP2-9, including xylobiose) (% dm)	39.5
Average DP of AXOS	5
A/X ratio of AXOS	0.19
Glucuronic acid bound to AXOS (% dm)	1.0
Ferulic acid bound to AXOS (% dm)	1.6
Glucose as part of poly/oligosaccharides	12.4
Galactose as part of poly/oligosaccharides	0.5
Mannose as part of poly/oligosaccharides	0.2
Total free monosaccharides	0.6
Protein (% dm)	0.6
Total fat (% dm)	< 0.5
Ash (% dm)	1.0
Dry matter (dm, %)	97.6

AXOS, arabinoxylan-oligosaccharides; A/X ratio, arabinose/xylose ratio; dm, dry matter; DP, degree of polymerization.

Supplementary Table S2: Primer sequences used for quantitative PCR

	Primer Forward	Primer Reverse
RPL19[a]	GAAGGTCAAAGGGAATGTGTTCA	CCTTGTCTGCCTTCAGCTTGT
MCP1[a]	GCAGTTAACGCCCCACTCA	CCCAGCCTACTCATTGGGATCA
IL1β[b]	TCGCTCAGGGTCACAAGAAA	CATCAGAGGCAAGGAGGAAAAC
IL6[b]	ACAAGTCGGAGGCTTAATTACACAT	TTGCCATTGCACAACTCTTTTC
F4/80b[b]	TGACAACCAGACGGCTTGTG	GCAGGCGAGGAAAAGATAGTGT
ZO-1[b]	TTTTTGACAGGGGGAGTGG	TGCTGCAGAGGTCAAAGTTCAAG
Occludin[b]	ATGTCCGGCCGATGCTCTC	TTTGGCTGCTCTTGGGTCTGTAT
Plag2g2[a]	AGGATCCCCCAAGGATGCCAC	CAGCCGTTTCTGACAGTTCTGG
Reg3g[a]	TTCCTGTCCTCCATGATCAAA	CATCCACCTCTGTTGGGTTC
Bifidobacterium spp.	GATTCTGGCTCAGGATGAACGC	CTGATAGGACGCGACCCCAT
Akkermansia muciniphila	CAGCACGTGAAGGTGGGGAC	CCTTGCGGTTGGCTTCAGAT

RPL19: Ribosomal protein L19; MCP1, monocyte chemoattractant protein 1; IL1β, interleukin 1 beta ; IL6, interleukin 6 ; F4/80, EGF-like module-containing mucin-like hormone receptor-like1 ; ZO-1, Zonula occludens 1; Plag2g2, phospholipase A2 group-II; Reg3g, regenerating islet-derived protein 3 gamma.

[a]primers from Everard, A. et al. Microbiome of prebiotic-treated mice reveals novel targets involved in host response during obesity. ISME J 8, 2116-2130; [b]primers from Cani, P. D. et al. Changes in gut microbiota control inflammation in obese mice through a mechanism involving GLP-2-driven improvement of gut permeability. Gut 58, 1091-1103,

Primers for *Akkermansia muciniphila* from "Derrien, M. 2007. Mucin utilisation and host interactions of the novel intestinal microbe *Akkermansia muciniphila*". Ph.D. thesis (ISBN 978-90-8504-644-8). Wageningen University, Wageningen, The Netherlands.

Primers for *Bifidobacterium* spp.: A RDP seqmatch search revealed that, among 335 hits over 307935 total searched, 335 hits belong to the *Bifidobacteriaceae*, with 324 being assigned to the genus *Bifidobacterium* (over 779 total searched), giving a specificity of 97% and a coverage of 42%. RDPseqmatch was used with the following dataset options: Strain both; Source Isolates; Size >1200; Quality Good; Differences Allowed 0.

Supplementary figure S1. Short chain fatty acids in the cecal content

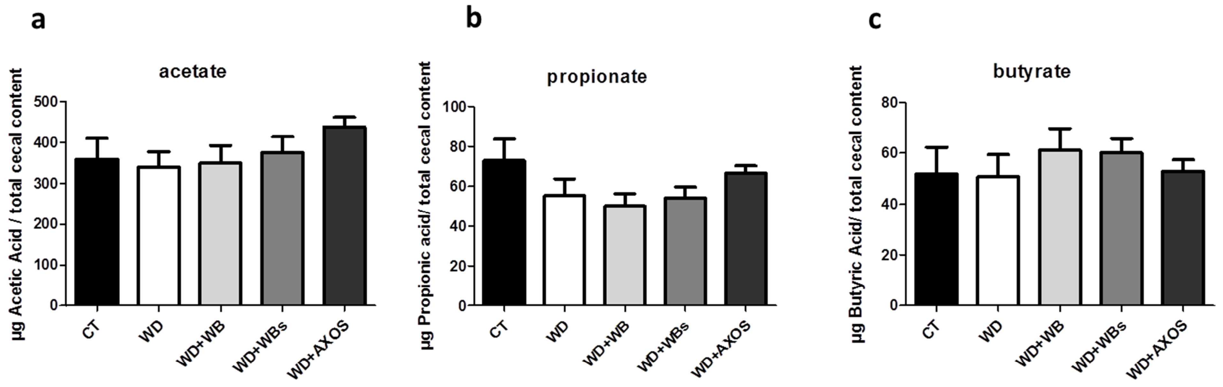

Acetate (**a**), propionate (**b**) and butyrate (**c**) in the cecal content of mice fed a control diet (CT), a western diet (WD), a WD supplemented with 5% of wheat bran (WD+WB), a WD supplemented with 5% of wheat bran with reduced particle size (WD+WBs) or a WD supplemented with 5% of AXOS (WD+AXOS) for 8 weeks. Results are expressed as mean ± SEM (n=7-9; ANOVA p>0.05).

Supplementary figure S2. Impact of the three wheat bran fractions on alpha-diversity indexes for richness and evenness.

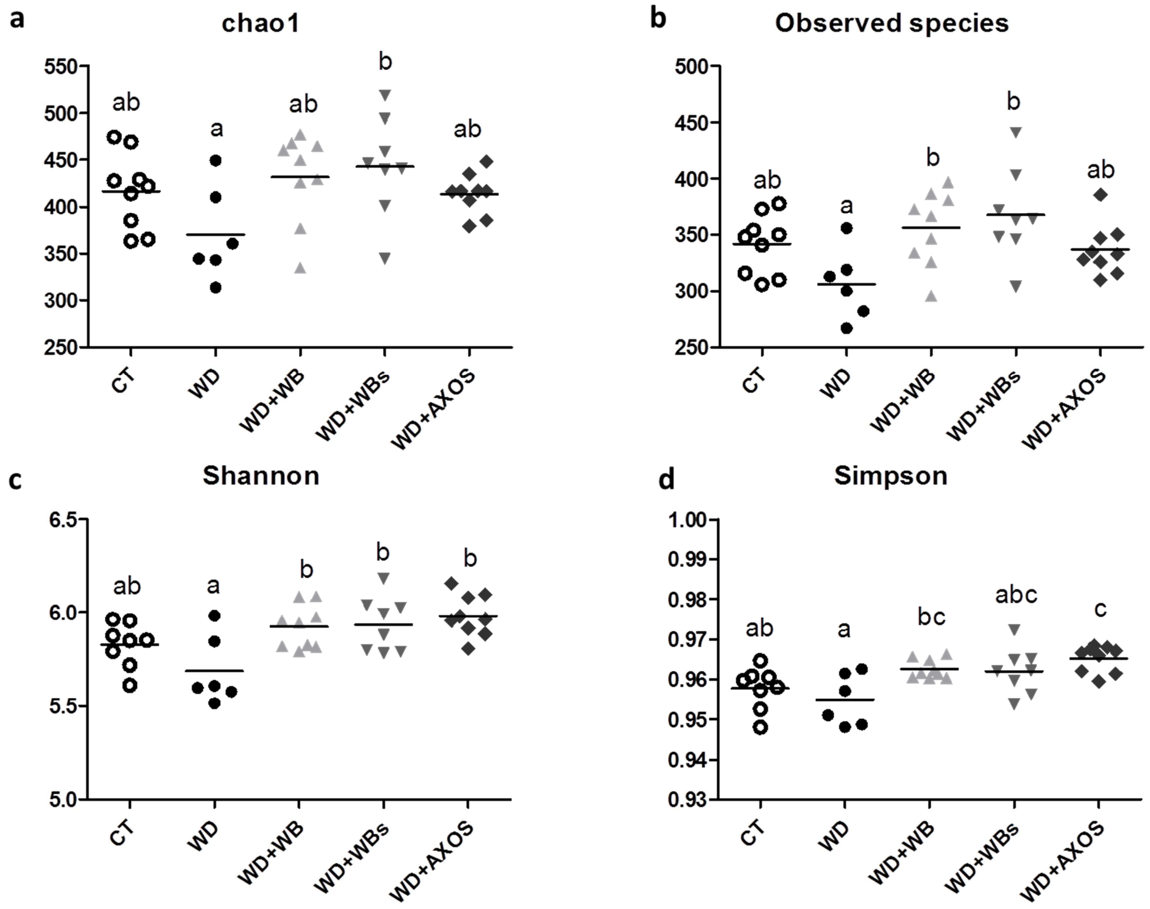

Diversity indexes for richness (chao1 and observed species, **a** and **b**). Diversity indexes for evenness (Shannon and Simpson indexes, **c** and **d**). Mice were fed a control diet (CT), a western diet (WD), a WD supplemented with 5% of wheat bran (WD+WB), a WD supplemented with 5% of wheat bran with reduced particle size (WD+WBs) or a WD supplemented with 5% of AXOS (WD+AXOS) for 8 weeks. Individual values are presented with mean. Data with different superscript letters are significantly different at $p < 0.05$ (ANOVA).

Supplementary figure S3. Impact of the three wheat bran fractions on the gut microbiota composition.

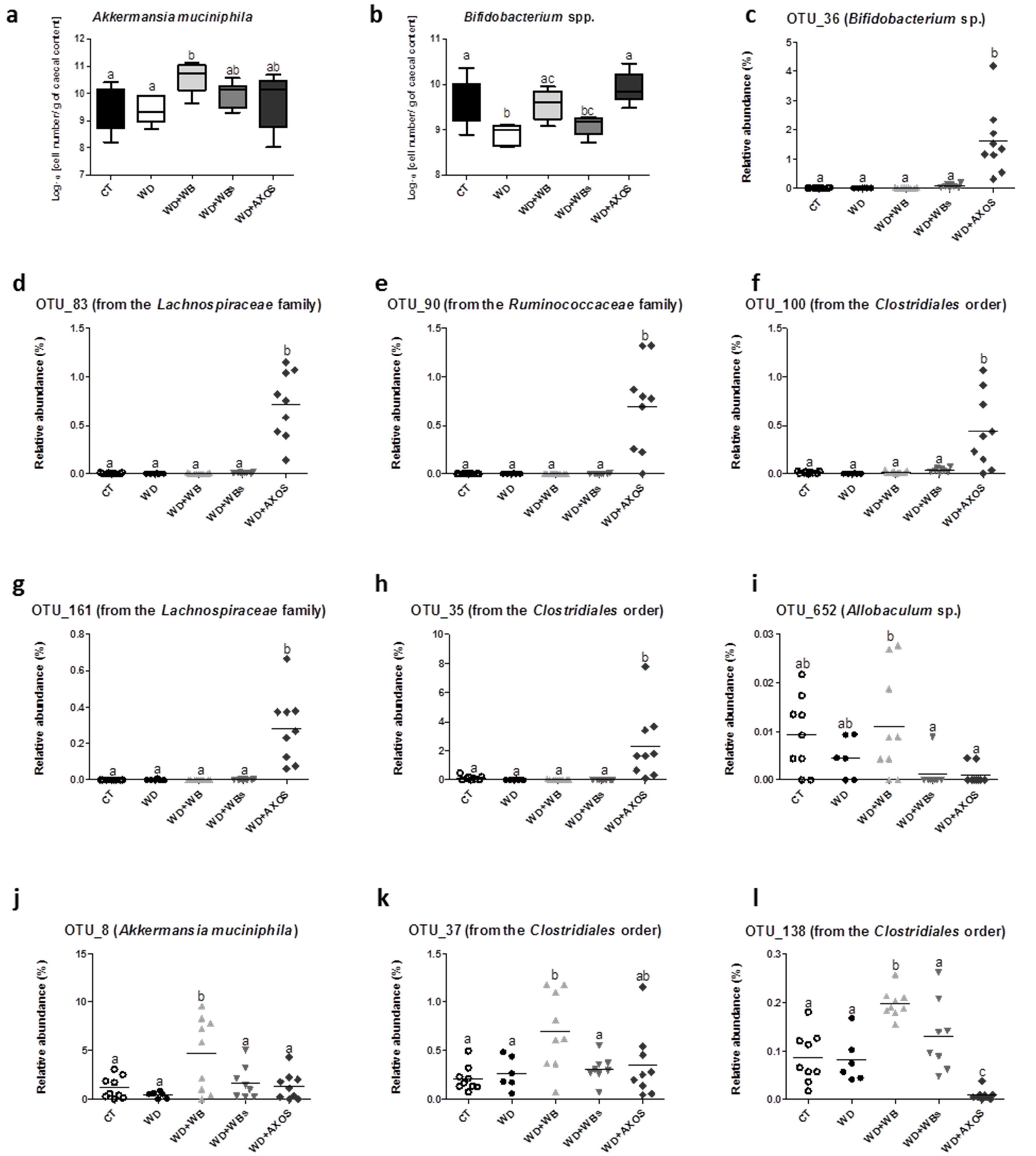

qPCR analyses of *Akkermansia muciniphila* (**a**) and *Bifidobacterium* spp. (**b**). Six OTUs were increased more than 100-fold upon AXOS (**c-h**). Two OTUs that were differentially impacted by the wheat bran fractions (**i,j**). The only 3 OTUs that were affected by WB but not by WBs (**j,k,l**). Mice were fed a control diet (CT), a western diet (WD), a WD supplemented with 5% of wheat bran (WD+WB), a WD supplemented with 5% of wheat bran with reduced particle size (WD+WBs) or a WD supplemented with 5% of AXOS (WD+AXOS) for 8 weeks.

Results are expressed as mean $\pm$ SEM (for **a**,**b**) or individual values are presented with mean (for **c-l**). Data with different superscript letters are significantly different at $p < 0.05$ (n=7-9; ANOVA).

Supplementary figure S4. Impact of cereal fractions on peptides assessed by Luminex technology.

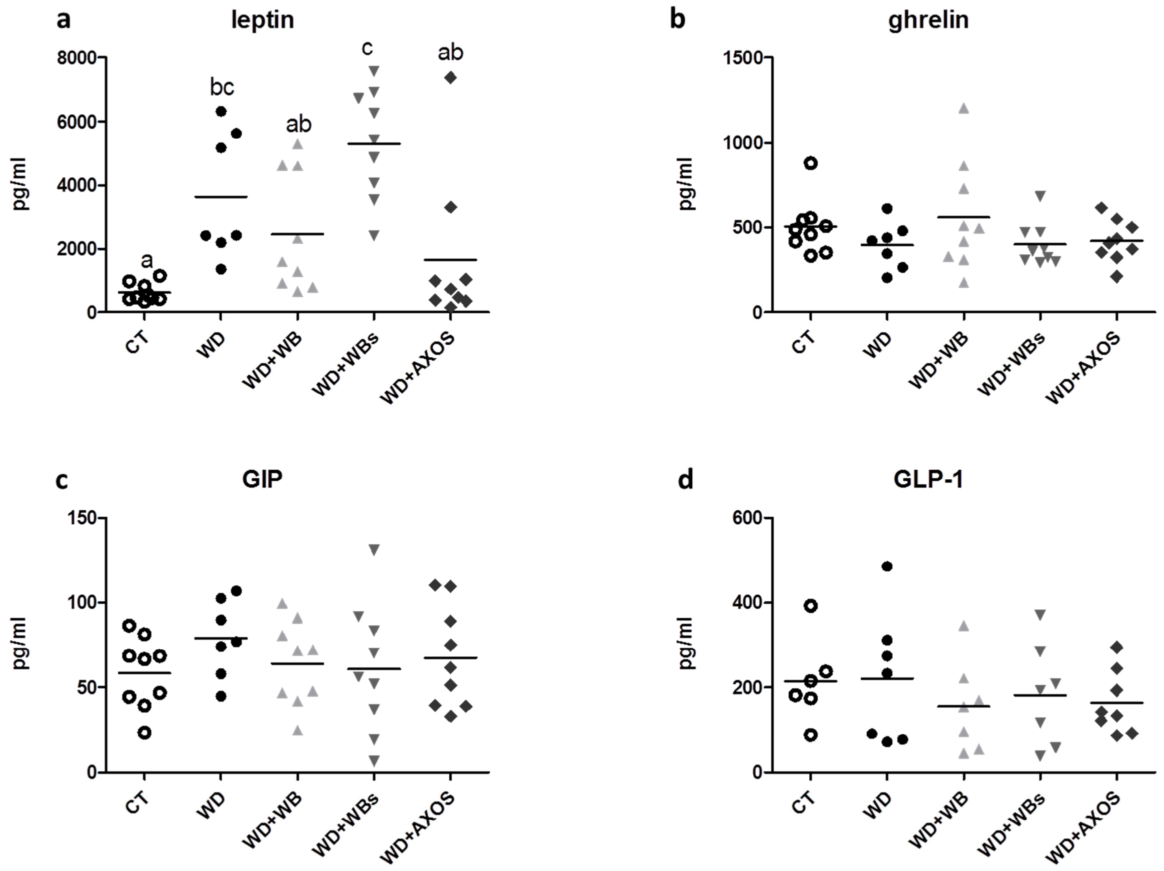

Portal concentrations of leptin (**a**), ghrelin (**b**), Glucose-dependent insulinotropic peptide (GIP) (**c**) and glucagon-like peptide-1 (GLP-1) (**d**). Mice were fed a control diet (CT), a western diet (WD), a WD supplemented with 5% of wheat bran (WD+WB), a WD supplemented with 5% of wheat bran with reduced particle size (WD+WBs) or a WD supplemented with 5% of AXOS (WD+AXOS) for 8 weeks. Individual values are presented with mean. Data with different superscript letters are significantly different at $p < 0.05$ (ANOVA).
